# Antioxidant Activity and Chemical Alterations of Honeybee Brood Bio-Peptides Interacting with Honey Under Moist-Dried Thermal Aging

**DOI:** 10.3390/antiox14030254

**Published:** 2025-02-21

**Authors:** Supakit Chaipoot, Pairote Wiriyacharee, Pattavara Pathomrungsiyounggul, Gochakorn Kanthakat, Chalermkwan Somjai, Kongsak Boonyapranai, Sirasit Srinuanpan, Worachai Wongwatcharayothin, Rewat Phongphisutthinant

**Affiliations:** 1 Multidisciplinary Research Institute, Chiang Mai University, Chiang Mai 50200, Thailand; supakit.ch@cmu.ac.th; 2 Center of Excellence in Microbial Diversity and Sustainable Utilization, Chiang Mai University, Chiang Mai 50200, Thailand; pairote.w@cmu.ac.th (P.W.); sirasit.s@cmu.ac.th (S.S.); 3 Faculty of Agro-Industry, Chiang Mai University, Chiang Mai 50100, Thailand; pattavara.p@cmu.ac.th (P.P.); gochakorn.g@gmail.com (G.K.); 4 Processing and Product Development Factory, The Royal Project Foundation, Chiang Mai 50100, Thailand; 5 Research Institute for Health Sciences, Chiang Mai University, Chiang Mai 50200, Thailand; kongsak.b@cmu.ac.th; 6 Department of Biology, Faculty of Science, Chiang Mai University, Chiang Mai 50200, Thailand; 7 Faculty of Humanity, Chiang Mai University, Chiang Mai 50200, Thailand; worachai.w@cmu.ac.th

**Keywords:** honeybee brood, honey, bio-peptides, antioxidant activity, moist-dried heating process, Maillard reaction

## Abstract

Edible insect honeybee brood and natural honey are rich in proteins and saccharides, with inherent bioactive properties such as antioxidant activity. To enhance their antioxidative potential under simple thermal conditions, this research employed spontaneous aging via a moist-dried heating process, primarily driven by the Maillard reaction. Honeybee brood bio-peptides (HBb-BPs), produced through *Rhizopus oligosporus* fermentation, were mixed with honey in varying ratios of 70:30, 50:50, and 30:70 (%*w*/*w*). The mixtures underwent interaction under controlled conditions (60 °C for 20 days at ~75% relative humidity). A comparative analysis was performed on the mixtures before and after the thermal interaction, focusing on chemical characterization and antioxidant activity (ABTS, DPPH, and FRAP assays). Results revealed that the post-process mixtures exhibited significantly enhanced antioxidant activity, with higher honey concentrations correlating to greater antioxidative effects. Furthermore, allulose and mannose were detected after processing, while levels of fructose, glucose, and free amino acids decreased. These changes likely indicate the formation of complex compounds, molecular rearrangements, and the production of phenolic compounds that contributed to the increased antioxidative capacity. This study highlights the pivotal role of the Maillard reaction in augmenting antioxidant activity, elucidates changes in sugar–amino acid interactions, and validates the effectiveness of the moist-dried heating process. These findings provide valuable insights for potential future applications of this simple and scalable method.

## 1. Introduction

Honeybees and their derivatives have played significant roles in culinary and therapeutic practices throughout history, as documented in ancient literature [1]. Over the centuries, humans have expanded the use of honey, recognizing its versatility beyond its traditional roles in its raw form and as a bee-derived product. Honey, as a natural sweetener, is primarily composed of carbohydrates (60–85%), with a moisture content of 12–23%, alongside minor quantities of proteins, amino acids, minerals, organic acids, vitamins, phenolics, and flavonoids [2,3,4,5]. In Thailand, particularly in the northern regions, longan honey is highly valued for its unique taste and aroma, derived from the longan fruit plant. It is widely used as a food ingredient, a beverage sweetener, and in traditional medicine [6]. Beyond honey itself, other honeybee-derived products such as bee pollen, bee bread, royal jelly, propolis, bee venom, and bee brood have demonstrated functional benefits across a broad spectrum of applications [7,8]. Honeybee brood (*Apis mellifera* L.) is recognized as a viable edible insect and has been traditionally consumed in numerous countries worldwide, including Mexico, Ecuador, Senegal, China, Zambia, Australia, and Thailand [9]. The optimal stage for harvesting honeybee brood larvae and pupae for consumption is typically 10 to 14 days into the drone development stage, marked by the pink coloration of the pupae’s eyes, which ensures both a palatable taste and maximum biomass yield [9,10]. Studies on honeybee brood reveal its remarkable nutritional profile, boasting high levels of protein, amino acids, fatty acids, carbohydrates, minerals, and vitamins, comparable in both quality and quantity to those found in beef protein [1,11,12,13].

Fermented protein foods derived from edible filamentous fungi have been extensively studied and developed alongside various sustainable protein sources such as algae, edible insects, cultured meat, and plant-based meat analogs [14]. Numerous fungal species have been employed in the fermentation of protein-rich substrates, including both meat and plant-based items such as cheese, sausages, dried-cured ham, preserved fish, air-dried meat, soy sauce, and tempeh. Edible filamentous fungi are particularly valued for their high protein content, quality, and digestibility. They also exhibit the capability to produce single-cell protein, commonly referred to as mycoprotein, suitable for human consumption. Moreover, they provide complex enzymes that enhance the ripening process, organoleptic properties, and bioactivity of the fermented products, making them a promising alternative nutritional source for human health [14,15,16]. The genus *Rhizopus*, especially *Rhizopus oligosporus*, is widely utilized in the production of tempeh-type products through solid-state fermentation, using legume seeds or cereal grain mixtures as the primary substrate. This genus is recognized as Generally Regarded as Safe (GRAS) by the Food and Agriculture Organization of the United Nations, particularly for its application in soy tempeh production in Asian countries [17]. During fermentation, the macromolecules in the substrate, including carbohydrates, proteins, and lipids, undergo partial decomposition and biotransformation. This process leads to the synthesis of bioactive compounds and the reduction of certain antinutritional factors, such as phytic acid, which is commonly found in beans, nuts, and grains and exhibits antinutrient effects that hinder mineral absorption [18,19,20]. A study by Zhang et al. [20] demonstrated that *R. oligosporus* RT-3 exhibits high levels of phytase activity capable of hydrolyzing phytic acid. In addition to phytase, *R. oligosporus* displayed a diverse range of enzymatic activities during solid-state fermentation, including protease, α-amylase, β-glucosidase, esterase, and cellulase. These enzymes catalyze the hydrolysis of substrate biomacromolecules into smaller bioactive molecules. As a result, the fermented products are enriched with bioactive components such as phenolic compounds, vitamins, and fatty acids, as well as amino acids like proline, tyrosine, tryptophan, histidine, and methionine. These enhancements significantly improve the antioxidant activity of the soybean-based products fermented with *R. oligosporus* [17,20].

In addition to fungal proteolysis, which produces bioactive peptides and amino acid sequences with antioxidant effects [21], the Maillard reaction plays a key role in enhancing antioxidant properties through the formation of sugar–peptide and/or sugar–amino acid interactions. This non-enzymatic browning reaction, particularly accelerated by thermal processes under various conditions such as cooking, baking, roasting, spontaneous aging, and incubation under moist-dried or wet heat conditions, leads to the formation of bioactive macromolecules, including Maillard reaction products and glycated protein–polysaccharide compounds. These compounds are formed through covalent interactions between the amine groups of proteins and the carbonyl groups of sugars. The products of this reaction exhibit enhanced bioactivity, especially in terms of antioxidative efficacy [22,23,24,25]. Moreover, the structural rearrangement or grafting of these compounds during the Maillard reaction also improves their techno-functional properties, making them valuable for various applications in food and bioprocessing industries [26,27,28].

Given the limited available information and recent interest in honeybee brood bio-peptides (HBb-BPs) produced through *Rhizopus oligosporus* fermentation, this study aims to investigate the chemical characterization and antioxidant activity of these bio-peptides when interacting with honey at varying concentrations under controlled moist-dried heating conditions, compared to untreated processes. In this context, HBb-BPs act as the protein source, and honey serves as the sugar source, both critical components in driving the Maillard reaction. This study is designed to identify the optimal proportion of these components for interaction via the Maillard reaction, exploring how variations in protein or sugar concentrations influence changes in chemical composition and bioactivity. Specifically, it evaluates the levels of sugars (fructose, glucose, mannose, and allulose), amino acid profiles, available free amino acid groups, degree of glycation, peptide molecular weight distribution, and changes in antioxidative properties. These findings are expected to provide insights into the chemical alterations associated with the enhanced antioxidative properties resulting from the Maillard reaction and structural rearrangement of peptide and saccharide molecules. Additionally, principal component analysis (PCA) was applied to investigate and validate correlations between different HBb-BP and honey treatments, both before and after the interaction process, in relation to their chemical and antioxidant responses.

## 2. Materials and Methods

### 2.1. Materials

Honeybee brood (*Apis mellifera* L.) in the larval to pre-pupal stages, similar to the description by Supakit et al. [29], was collected from a Longan honey farm in Maewang District, Chiang Mai Province, Thailand. The raw brood was steamed for 1 h using a stainless-steel steamer and then stored in plastic bags in a freezer until further use. The honey used in this study, derived from the longan (*Dimocarpus longan* Lour.) plant and referred to as longan honey, was obtained from Koonton Bee Farm in Sansai District, Chiang Mai Province, Thailand. The sugar composition of the honey consisted of fructose (36.12 ± 0.40%) and glucose (31.65 ± 0.34%). The *Rhizopus oligosporus* strain TISTR3527 was obtained from the Biodiversity Research Centre (BRC) of the Thailand Institute of Scientific and Technological Research (TISTR) in Pathum Thani, Thailand. These materials were used as raw ingredients in the research.

### 2.2. Honeybee Brood Protein Extraction and Honeybee Brood Bio-Peptides (HBb-BPs) Preparation

The extraction of honeybee brood protein began by mixing and blending raw steamed honeybee brood with deionized water at a ratio of 1:3 (*w*/*v*) for 1 min followed by filtration through a 10 µm nylon bag to obtain a solution of honeybee brood. The pH of the solution was then adjusted to 8 using a sodium hydroxide solution, and the mixture was subsequently centrifuged at 7500 rpm at 4 °C for 10 min, resulting in the collection of the honeybee brood precipitate. This precipitate was then subjected to fat content removal through agitation with hexane in a 1:10 (*w*/*v*) ratio for 3 h. After shaking, the hexane was separated from the honeybee brood sediment through filtration. The sediment was then dried overnight at room temperature, yielding honeybee brood protein powder with a total protein content of 73.42 ± 0.28%.

Honeybee brood bio-peptides (HBb-BPs) were produced by hydrolyzing honeybee brood protein powder using a myco-fermentation method. Initially, the honeybee brood protein was adjusted to a moisture content of ~45–50% [30] and then sterilized at 115 °C in an autoclave for 15 min. Following autoclaving, the protein was inoculated with approximately 10% of a starter culture of *R. oligosporus* (10^6^ spores/mL) for solid-state fermentation and incubated at 25 °C for 48 h. Subsequent to fermentation, the product was subjected to drying via freeze drying, followed by grinding to achieve a powdered form of HBb-BPs. This powdered sample was stored at −18 °C in a polyethylene bag pending its utilization in subsequent experiments.

### 2.3. Interaction of Honeybee Brood Bio-Peptides (HBb-BPs) with Honey Using the Moist-Dried Heating Method with Spontaneous Aging

A factorial experiment was carried out using a completely randomized design (CRD) to assess the effects of spontaneous thermal interaction between HBb-BPs and honey. The interaction was achieved by mixing different amounts of HBb-BPs and honey in three concentration ratios: 70:30, 50:50, and 30:70 by mass percentage. The Maillard reaction was initiated using a moist-dried heating method, where the mixture was aged in a desiccator under controlled conditions of 75% relative humidity and 60 °C for 20 days. After aging, the samples were frozen at −18 °C and then analyzed for chemical and antioxidant properties.

### 2.4. Chemical Characteristics Determination of Honeybee Brood Bio-Peptides Interaction with Honey

#### 2.4.1. Proximate Analysis for HBb-BPs and Honey

The raw materials of HBb-BPs and honey were analyzed using AOAC methods, as referenced by Finke [11] and Chua and Adnan [31]. This thorough analysis involved determining the moisture, protein, fat, carbohydrate, fiber, and ash content in the samples.

#### 2.4.2. Monosaccharides Profile by HPLC

The monosaccharide profile was analyzed using High-Performance Liquid Chromatography (HPLC) [23] with a polymer-based Hydrophilic Interaction Liquid Chromatography (HILIC) column (HILICpak VG-50 4E, Showa Denko, Tokyo, Japan), which had an internal diameter of 4.6 mm and a length of 250 mm. The analysis was performed with a refractive index detector (Shimadzu, Kyoto, Japan). The mobile phase was a mixture of acetonitrile, methanol, and deionized water in a ratio of 85:10:5 (*v*/*v*). The operational conditions included a flow rate of 0.6 mL/min, a column temperature of 50 °C, and a total run time of 45 min. Prior to injection, samples were dissolved in a 50% acetonitrile solution and filtered through a 0.20 µm membrane filter. A 10 µL sample was injected into the column, and the concentration of each sugar was determined by analyzing the retention time and constructing a linear plot of peak areas corresponding to various sugar concentrations.

#### 2.4.3. Amino Acids Profile Analysis

The quantification of seventeen amino acids was performed using the post-column derivatization technique [23,32]. A column with a stationary phase composed of Na-type sulfone groups, a particle size of 5 µm, and dimensions of 6 mm I.D. × 100 mm was used for the separation of mixed standard amino acid solutions and samples (Shimadzu, Japan). Three mobile phases were used for amino acid analysis: citrate buffer at pH 3.23 (Mobile Phase A), citrate buffer at pH 10.00 (Mobile Phase B), and a 0.2 M NaOH solution (Mobile Phase C). The OPA/N-acetylcysteine reaction reagents were prepared to react with amino acids in the sample after separation from the column. The reaction took place in the reaction coil stage, forming fluorescent derivatives, which were subsequently detected using an RF detector. The sample analysis was conducted under fixed conditions, with the mobile phase flow rate set at 0.4 mL/min, the column oven temperature maintained at 60 °C, and a retention time of 65 min. Samples were prepared by dissolving them in a diluent solution (pH 2.2) [25] and then filtered through a 0.45 µm syringe filter before injecting a volume of 10 µL. Amino acid quantification was performed by analyzing retention times and the linear correlation of peak areas across different concentrations. The results were then converted from micromole units to milligrams per 100 g on a dry weight basis.

#### 2.4.4. Peptide Length and Molecular Weight Distribution Using Size-Exclusion HPLC

Size-exclusion chromatography (SEC or SEC-HPLC) was employed to analyze the molecular weight of soluble peptides in the samples and estimate their relative molecular weight distribution [25]. The analysis was conducted using a Sepax SRT-C SEC-300 gel filtration column with a hydrophilic polymer phase, a 5 µm particle size, and dimensions of 300 mm × 7.8 mm (length × I.D.). A 0.1 M sodium phosphate buffer (pH 7) was used as the mobile phase under isocratic elution. Detection was performed at 280 nm using a photodiode array detector or a UV-VIS detector. The mobile phase flow rate was set at 1 mL/min at 30 °C. Sample injections of 15 µL were prepared by dissolving the sample in water and filtering it through a 0.45 µm filter. Peptide molecular weight markers ranging from 15 kDa to 600 kDa were used, consisting of various peptide sizes, including p-aminobenzoic acid (pABA, ~137 Da), ribonuclease A type I-A (~13.7 kDa), ovalbumin (~44.3 kDa), gamma-globulins (~150 kDa), and bovine thyroglobulin (~670 kDa). A logarithmic curve plotting the molecular weight of peptide markers against retention time was constructed to derive an equation for estimating the relative peptide size distribution, incorporating chromatogram area integration.

#### 2.4.5. Free Amino Acid Groups and Degree of Glycation by OPA Assay

The free amino acid group was quantified using a modified OPA assay [33], which focused on measuring the loss of free amino groups. The OPA reagent was prepared by dissolving 0.2 g of OPA in 5 mL of absolute ethanol, followed by the addition of 125 mL of 0.1 M sodium tetraborate buffer (pH 9.75), 0.5 mL of β-mercaptoethanol, and 12.5 mL of 10% (*w*/*v*) sodium dodecyl sulfate (SDS). The mixture was then diluted to a final volume of 250 mL with distilled water. Approximately 50 µL of the sample was mixed with 3 mL of the OPA reagent at 25 °C and incubated for 2 min. The absorbance of the samples was measured at 340 nm, and a standard curve of lysine concentration (mg lysine per 100 g) was used for quantification [25].

The degree of glycation (DG) was calculated using the formula in Equation (1), where A0 represents the initial absorbance of the sample before the interaction process, and A20 days denotes the absorbance after the 20-day interaction period for each treatment.

DG (%) = (A_0_ − A_20 days_)/A_0_) × 100 (1)

#### 2.4.6. Total Phenolic and Flavonoid Compounds Determination

These analyses were modified from methods used by Pauliuc et al. [34] and Sawicki et al. [35]. Approximately 1 g of the HBb-BPs sample was extracted using 10 mL of 80% methanol and stirred with a magnetic stirrer for 15 min at room temperature in the dark. The mixture was then centrifuged at 7500 rpm for 10 min to obtain the supernatant extract. For the total phenolic content (TPC) test, 200 μL of the extract was combined with 2 mL of Folin–Ciocalteu reagent (diluted 1:10 with water) and 1.8 mL of 7.5% Na_2_CO_3_ solution. The mixture was incubated in the dark for 20 min, and the absorbance was measured at 750 nm using Gallic acid as the standard to create a calibration curve with a UV spectrophotometer.

The same extract was also used to measure total flavonoid content (TFC). Approximately 5 mL of the extract was mixed with 300 μL of 5% NaNO2 solution and 300 μL of 10% AlCl_3_ solution, vortexed, and kept in the dark for 5 min. Then, 2 mL of 1 M NaOH was added, and the mixture was allowed to sit for 6 min before measuring absorbance at 510 nm, using Quercetin as the standard. Both TPC and TFC were expressed per 100 g of the dry sample.

#### 2.4.7. Antioxidative Properties of HBb-BPs Interaction with Honey

Each sample containing HBb-BPs mixed with honey was evaluated for antioxidant activity using three different methods: the ABTS radical cation assay, DPPH radical scavenging activity, and ferric reducing antioxidant power (FRAP), as described by Chaipoot et al. [24] and Phongphisutthinant et al. [25].

ABTS assay: A mixture was prepared by combining 2.45 mM potassium persulfate with 7 mM ABTS stock solution and 20 mM sodium acetate buffer (pH 4.5). This mixture was then incubated in the dark at room temperature for 12–16 h. Before use, the ABTS reagent was diluted with 95% ethanol to achieve an absorbance of 0.70 ± 0.02 at 734 nm. The sample solution was mixed with 2 mL of ABTS reagent, followed by agitation and incubation in the dark for 6 min before measuring absorbance at 734 nm using a spectrophotometer (UV1800; Shimadzu, Japan). The inhibition of ABTS radicals was assessed using a Trolox calibration curve, with results expressed as milligrams of Trolox equivalents (TEs) per 100 g of the sample.

DPPH assay: A 0.2 mM DPPH solution was prepared daily in 80% methanol. 1 mL of the sample was mixed with 2 mL of this DPPH solution, stirred well, and left to incubate at room temperature in the dark for 30 min. Absorbance was then measured at 517 nm using a UV–visible spectrophotometer. A control sample was prepared following the same procedure, replacing the sample with distilled water. A Trolox calibration curve was used to quantify the antioxidant activity in milligrams of Trolox equivalents (TEs) per 100 g of the sample.

FRAP assay: A reagent solution was prepared daily by mixing 2.5 mL of 10 mM TPTZ (2,4,6-tripyridyl-s-triazine) solution in 40 mM HCl, 2.5 mL of 20 mM FeCl_3_·6H_2_O solution, and 20 mL of 300 mM acetate buffer (pH 3.6). The mixture was incubated at 37 °C for 30 min, after which 50 μL of the sample was combined with 750 μL of the FRAP solution and incubated in the dark for another 30 min. The color change was measured at 593 nm, and a standard curve was established using FeSO_4_·7H_2_O. The results were reported in milligrams of FeSO_4_ equivalents per 100 g of the sample.

### 2.5. Statistical Analysis

Statistical analyses were carried out using SPSS software version 17.0 (SPSS Inc., Chicago, IL, USA). A one-way analysis of variance (ANOVA) was performed to assess significant differences, followed by Tukey’s multiple comparisons test. A significance level of *p* ≤ 0.05 was considered. Additionally, a biplot of principal component analysis (PCA) was created to visualize the effects or correlations of each treatment on various chemical characteristics and antioxidant properties. SPSS was also used to generate a PCA biplot to illustrate the impact or relationship of each treatment with the chemical and antioxidant properties.

## 3. Results and Discussion

### 3.1. Proximate Composition of Honeybee Brood Bio-Peptides (HBb-BPs) and Honey

The two raw materials, HBb-BPs and honey, were subjected to proximate composition analysis, as summarized in Table 1. HBb-BPs served as the primary protein source, with a protein content of 81.57 g/100 g on a dry weight basis and a water content of 42.15 g/100 g. Minor amounts of ash, fiber, fat, and carbohydrates were detected, with values of 6.27 g/100 g, 5.81 g/100 g, 3.60 g/100 g, and 2.75 g/100 g, respectively. On the other hand, honey, serving as the carbohydrate source, exhibited a carbohydrate content of 99.24 g/100 g on a dry weight basis, with a moisture content of 11.06 g/100 g. Trace amounts of protein, ash, and fat were present at 0.54 g/100 g, 0.19 g/100 g, and 0.03 g/100 g, respectively, with no detectable fiber content. Both raw materials had a caloric value of 369.65 kcal/100 g for HBb-BPs and 399.40 kcal/100 g for honey.

According to Rutka et al. [10], lyophilized drone broods were found to contain protein ranging from 32.0 to 52.4 g/100 g, carbohydrates from 17.8 to 38.9 g/100 g, fats from 21.1 to 24.2 g/100 g, and ash from 2.7 to 4.1 g/100 g. Factors such as bee species, stage of honeybee brood development, geographical location, and climate were identified as significant influences on the composition of bee broods. Additionally, Lima et al. [17] demonstrated a significant increase in protein content following *R. oligosporus* growth during the solid-state fermentation of cashew byproducts, leading to the formation and incorporation of mycoprotein. Similarly, in the present study, HBb-BPs exhibited a 1.11-fold increase in protein content compared to the honeybee brood protein extract before fermentation, with an average protein content of 73.42 g/100 g.

The proximate composition of general honey shows that its moisture content ranges from 12% to 23% [4,36]. For example, a specific honey type derived from longan nectar in Thailand was found to have a moisture content of 17.80% [6]. Honey carbohydrates typically comprise 60% to 80%, primarily consisting of fructose and glucose as monosaccharides. The protein content, originating from bee saliva, varies from 0.1% to 2.0%, depending on the plant pollen sources. Honey also contains approximately 0.04% to 0.27% fat, primarily due to residual wax from harvesting [2,4,37]. In honey from Anfilo District, Southwest Ethiopia, a minor level of ash ranging from 0.27 to 0.64 g/100 g was detected, which included trace minerals such as potassium, calcium, sodium, and copper [37]. Additionally, honey provides an approximate energy value of 300 kcal per 100 g, largely derived from its sugar content, which constitutes about 15% of the daily recommended energy intake [38].

### 3.2. Changes in Chemical Compositions Before and After Interaction with the Moist-Dried Heating Process Through Spontaneous Aging of Different HBb-BPs to Honey Ratios

#### 3.2.1. Changes in the Compositions of Monosaccharide

In addition to measuring eight types of sugars, four specific sugars (fructose, glucose, mannose, and allulose) were analyzed in each mixture sample of HBb-BPs combined with honey at different concentration levels (30%, 50%, and 70%), both before and after the moist-dried heating process, as shown in Table 2. Before the interaction process, all mixtures contained fructose and glucose, with no detectable levels of mannose and allulose. The amounts of fructose and glucose varied and increased depending on the amount of honey added to the mixture. Fructose levels ranged from 10.8 to 25.3 g/100 g, while glucose levels ranged from 9.2 to 22.9 g/100 g. After the interaction process, the fructose content showed a slight decrease, ranging from 4.8 to 23.8 g/100 g. In contrast, glucose levels decreased significantly following the moist-dried heating process, with remaining amounts ranging from 0.3 to 4.4 g/100 g. Consequently, the percentage of sugar loss varied for each sugar. Fructose exhibited losses of 55.56%, 31.49%, and 5.92% for the HBb-BPs-honey mixtures at 70:30, 50:50, and 30:70 ratios, respectively, while glucose content decreased by 96.74%, 95.95%, and 80.79%, respectively. However, mannose and allulose were detected after the interaction process, and their concentrations increased with higher honey concentrations. The levels ranged from 0.5 to 1.4 g/100 g for mannose and from 0.6 to 1.9 g/100 g for allulose.

Longan honey, a popular variety in Thailand, is primarily composed of fructose and glucose as monosaccharides. It also contains disaccharides and trisaccharides, such as palatinose, isomaltose, melibiose, melezitose, raffinose, and maltitol, among others, which account for approximately 1–2% of its composition. In addition, 18 other sugars have been identified through analysis [6]. Another review by Khan et al. [3] and Machado De-Melo et al. [4] reported that honey is composed of approximately 76.7% reducing sugars, with over 22 different types of sugars detected. Among these, fructose makes up 38.4%, glucose 30.3%, and sucrose 1.3%, while the remaining sugars consist of small quantities of disaccharides, trisaccharides, oligosaccharides, and polysaccharides.

As demonstrated in this study, the compositions of individual sugars in the mixture of HBb-BPs and honey underwent changes due to thermal processing after the moist-dried heating treatment. These changes are potentially related to the Maillard reaction and/or other reactions such as aldose–ketose isomerization (Lobry-de-Bruyn-Alberda-van-Ekenstein transformation, LA transformation) and enolization. Consequently, the Maillard reaction may lead to a reduction in fructose and glucose content in the samples due to interactions between reducing sugars and the amino groups of HBb-BPs. Additionally, the LA transformation may contribute to the production of allulose, with higher honey concentrations resulting in increased allulose formation. Similarly, the formation and increase of mannose were observed after the moist-dried heating processes, where glucose molecules may transform through enolization into mannose and fructose structures [33,39,40,41]. Allulose, also known as psicose or D-ribo-2-hexulose, is a rare sugar naturally found in small amounts in certain fruits, plants, and processed food products. The procedure of thermal processing at high temperatures, long storage, and high pH conditions can also promote allulose formation. It is formed through epimerization at the C-3 position of fructose [23,33,42,43]. Research conducted by Xie et al. [41] investigated honey from China and detected this sugar, which was identified as a new marker for adulteration in jujube honey. The average content of allulose in jujube honey was found to be 0.21%. Additionally, allulose was also detected in various syrup samples made from rice, cassava, wheat, corn, and other sources. Both mannose and allulose are bioactive monosaccharides with functional properties that offer potential health benefits. Mannose has been studied for its potential immune system support, as well as its effectiveness against metabolic syndrome, diabetes, intestinal diseases, and urinary tract infections [44]. Conversely, allulose has been associated with multiple physiological functions, including anti-inflammatory, anti-atherosclerosis, anti-obesity, anti-diabetes, anti-cancer, and antioxidant properties [45]. In cases where mannose is present in honey, it is considered a marker for detecting sugar adulteration in floral honey. While mannose is not commonly found in normal honey, its presence can be detected in honeydew honey, depending on the geographical area and flowering plants. Moreover, this sugar can also be observed in purified, high-end syrup products, mainly sourced from China and treated using ion exchange resins [39].

In addition, the raw materials utilized in this research were complex, necessitating an investigation into the structural arrangement, sequence characterization, and sequence-specific binding of these macromolecules. This requires the use of advanced and specific techniques provided by instruments such as high-resolution mass spectrometry (MS), X-ray diffraction (XRD), cryo-electron microscopy (cryo-EM), and heteronuclear single quantum coherence spectroscopy (HSQC), among others. These techniques can offer valuable insights into the composition and properties of these compounds, thereby revealing the techno-functional properties of these complex components [46,47].

#### 3.2.2. Changes in the Compositions of Amino Acid Profiling

All mixed samples of HBb-BPs interacting with various honey concentrations, both before and after the moist-dried heating process, were analyzed for the composition of seventeen types of amino acids. The total sum of these amino acids is presented in Table 3, along with the percentage of remaining amino acids. Higher honey concentrations resulted in lower amino acid levels before processing, with total amino acid contents of 1215.40 mg/100 g, 1003.89 mg/100 g, and 815.53 mg/100 g for 70%, 50%, and 30% HBb-BPs additions, respectively. Major amino acids detected in all mixtures, listed in descending order of content, included valine, alanine + cysteine, phenylalanine, and glutamic acid, while minor amounts were found for threonine, tyrosine, proline, leucine, histidine, lysine, serine, isoleucine, glycine, aspartic acid, methionine, and arginine.

After the moist-dried heating process for 20 days, the amino acid profiles of all samples changed significantly, with a notable decrease in the content of each amino acid. Specifically, the percentage of remaining amino acids, especially methionine, tyrosine, phenylalanine, and arginine, was undetectable in all mixed samples. Furthermore, three amino acids (threonine, isoleucine, and leucine) were no longer detected in the samples with 50% and 70% honey concentrations. In contrast, the level of valine showed only a slight decrease, with a higher proportion remaining (92.53% to 58.50%) compared to other amino acids, where the remaining content was below 50%. Overall, the total sum of amino acids in the mixed samples after the interaction process ranged from 151.36 to 423.72 mg/100 g, with remaining percentages of 13.42%, 15.88%, and 26.76% for HBb-BPs and honey ratios of 30:70, 50:50, and 70:30 (% *w*/*w*), respectively.

The reduction in amino acid content after thermal processing and prolonged storage may be attributed to the interaction between amino acids and reducing sugars, similar to the observed reduction in simple sugars from previous analyses. Notably, the addition of honey concentrations exceeding 50% resulted in a less than 20% decrease in remaining amino acid content after the interaction process, suggesting that higher sugar concentrations may limit the interaction between sugars and amino acids. This phenomenon is likely due to non-enzymatic browning or the Maillard reaction, leading to the formation of sugar-protein conjugates [22,23,24,25]. Another study by Kijewska et al. [47] found that the α-amino group of peptides reacts with lactose under dry heat or solid-phase conditions at high temperatures for a short duration. This reaction may result in the deamination of the N-terminal amino acid moiety, eventually leading to the formation of an α-ketoacyl derivative. Moreover, the Maillard reaction can generate a variety of flavors, such as caramel, nutty, and chocolate notes, depending on the composition and reaction pathways of amino acids and carbonyl compounds [48]. In fact, main Amadori compounds, such as N-(1-deoxy-1-fructosyl) phenylalanine (Fru-Phe) and N-(1-deoxy-1-fructosyl) proline (Fru-Pro), were detected in chaste honey after a storage period, following heat-induced Maillard reactions [49].

#### 3.2.3. Changes in Free Amino Acid Groups and Degree of Glycation

**Figure 1:**
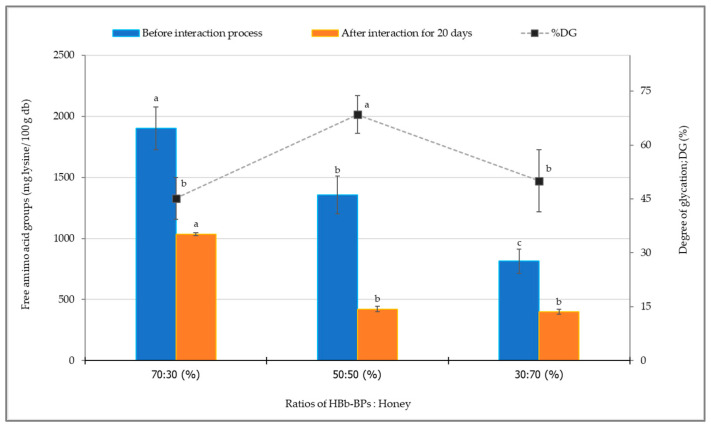
Free amino acid groups and degree of glycation before and after interaction through spontaneous aging for 20 days under a moist-dried heating process of HBb-BPs and honey at ratios of 70:30, 50:50, and 30:70 (% *w*/*w*). Different lowercase letters indicate significant differences among the different honey concentrations before and after the moist-dried heating process (*p* ≤ 0.05).

The availability of free amino acid groups (α- and ε-amino functions of HBb-BPs) and the degree of glycation were assessed using the OPA assay (Figure 1). Prior to the interaction process, all mixed samples of HBb-BPs with varying honey concentrations showed a decrease in free amino groups as the honey concentration increased. After 20 days of the interaction process, all samples exhibited a significant reduction in free amino acid groups. However, no significant difference was observed in the free amino acid groups between the samples of HBb-BPs mixed with honey at ratios of 50:50 and 30:70 (% *w*/*w*). When calculated as the degree of glycation, the results showed glycation degrees of 45.15%, 68.49%, and 50.04% for honey additions of 30%, 50%, and 70%, respectively. The highest degree of glycation was observed in the HBb-BPs mixed with honey at a 50:50 ratio, followed by the 30:70 and 70:30 ratios, which did not show a significant difference between them. This may be attributed to the limitations of peptide or sugar content in the treatment process involved in the Maillard reaction.

Dursun Capar and Yalcin [50] conducted a study on protein–saccharide conjugation via the Maillard reaction in a wet heating process. They found that optimizing the protein-to-saccharide ratio at 1:2, or approximately 70% saccharide content, achieved maximum conjugation efficiency and yield. Additionally, Ogutu et al. [51] observed that a 1:1 ratio, or 50% glucose interacting with benzylamine, was the most suitable concentration ratio for glycation under the Maillard reaction. The Maillard reaction, in its early stages, exhibits various pathways for forming complex components that contribute to beneficial properties such as free radical scavenging. These pathways include oxidation, acid hydrolysis, dehydration, enolization, structural fragmentation, and synergistic phenolic combinations [52,53,54]. Moreover, previous studies by these authors have highlighted the interaction of free amino groups with the carbonyl sites of carbohydrates during the moist-dried heating process. The covalent bonds formed in this reaction are linked to the Maillard reaction, while non-covalent bonds may also occur between phenolic compounds and amino acids. Both interactions likely contribute to the formation of complex components, resulting in higher antioxidant activity compared to amino acids or carbohydrates alone [23,24,25].

#### 3.2.4. Changes in Peptide Molecular Weight Distribution

**Figure 2:**
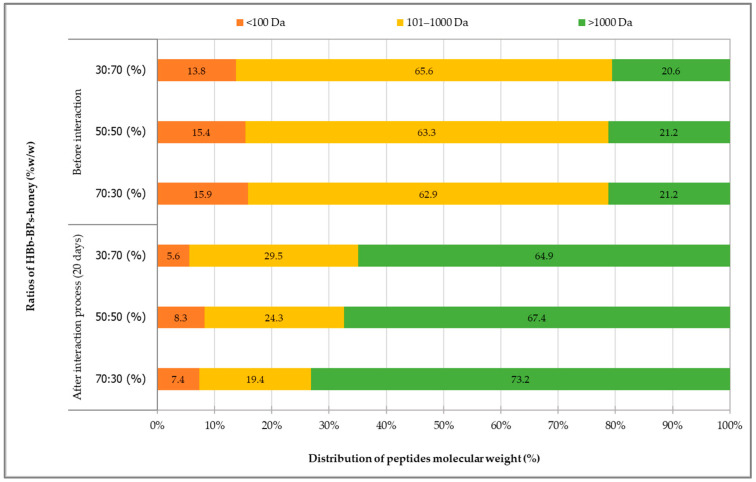
Distribution of peptide molecular weight, analyzed using HPLC, before and after interaction through spontaneous aging for 20 days under a moist-dried heating process of HBb-BPs and honey at ratios of 70:30, 50:50, and 30:70 (% *w*/*w*).

The distribution of peptide molecular weight percentages in all mixtures of HBb-BPs interacting with different concentrations of honey, both before and after the moist-dried heating process, is shown in Figure 2. The distribution was categorized into three groups based on the peptide sizes: less than 100 Da, 101–1000 Da, and greater than 1000 Da. Initial samples of HBb-BPs mixed with various honey concentrations revealed peptide chains with molecular weights of <100 Da, 101–1000 Da, and >1000 Da, averaging 13.8–15.9%, 62.9–65.6%, and 20.6–21.2%, respectively. However, after a 20-day interaction process, the mixtures exhibited changes in the distribution of peptide chain molecular weights. Peptides larger than 1000 Da increased in proportion, whereas peptides smaller than 1000 Da showed a reduction in percentage distribution compared to the samples before incubation. Higher honey concentrations resulted in a reduction in the size of peptide molecular weights, with the >1000 Da peptide range increasing to 64.9–73.2%. Conversely, medium-sized peptides (101–1000 Da) showed an increase in chain size, ranging from 19.4–29.5% of the distribution. Smaller peptides (<100 Da) were the most abundant in HBb-BPs mixed with honey at a 1:1 ratio.

Proteomic analysis conducted by Bong et al. [55] on Manuka honey revealed that aged honey undergoes chemical protein modifications, including interactions with phenolic compounds and/or the formation of Maillard reaction products. Peptide degradation may occur during storage, leading to a shift in the protein profile. Thermal processes involving prolonged incubation, such as the Maillard reaction or glycosylation between reducing sugars and peptides, could result in peptide degradation and rearrangement into more complex structures with higher molecular weight fractions [25,56]. Although there is limited information on honeybee brood peptides, some studies have been conducted on bee larvae plasma, revealing peptide molecular weight bands ranging from 3.5 to 260 kDa [57]. In contrast, honey and royal jelly have been investigated for their peptide and glycoprotein profiles. Two polypeptides with sizes around 29–61 kDa were identified in honey [58], while royal jelly was found to contain a main peptide band around 5 kDa and minor bands around 10 kDa [59]. Studies on acacia and ziziphus honey also revealed the presence of short and cyclic peptide structures, which may have potent medical benefits for various bodily systems, including the immune, digestive, cardiovascular, and endocrine systems [60]. The alteration factors of honey concentration and incubation in this study may contribute to an increase in high-molecular-weight peptides while reducing smaller peptides. These changes align with protein modifications observed in aged honey, potentially due to glycosylation and Maillard reactions. Such transformations may enhance honey’s bioactivity; however, further investigation is required to understand their functional and health implications.

### 3.3. Changes in Total Phenolic and Flavonoid Content Before and After the Moist-Dried Heating Process of HBb-BPs Interacting with Different Honey Concentrations

**Figure 3:**
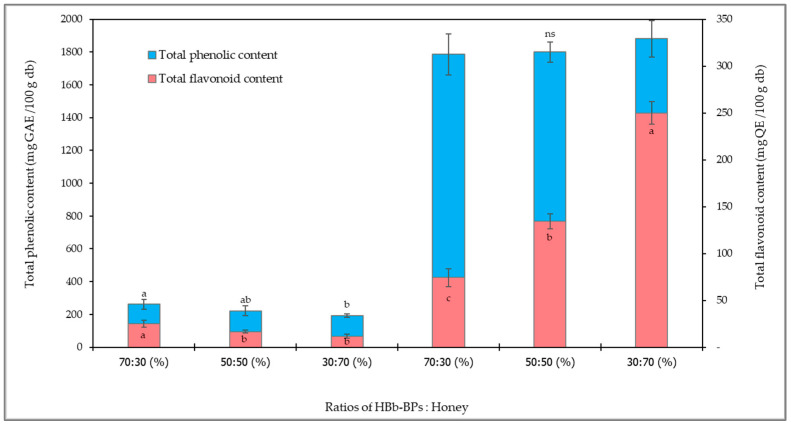
Total phenolic and total flavonoid content of HBb-BPs and honey at ratios of 70:30, 50:50, and 30:70 (% *w*/*w*) before and after interaction through spontaneous aging for 20 days under a moist-dried heating process. Different lowercase letters indicate significant differences among the different honey concentrations before and after the moist-dried heating process (*p* ≤ 0.05). ns indicates no significant difference.

As shown in Figure 3, the total phenolic and flavonoid content were determined for all samples of HBb-BPs mixed with three different concentrations of honey. The results indicated that, before the interaction process, both the total phenolic and flavonoid content decreased as the quantity of honey increased. However, no significant difference in phenolic and flavonoid content was observed between the 50:50 and 30:70 ratios of HBb-BPs to honey. The phenolic content ranged from 193.28 to 261.87 mg Gallic acid equivalent/100 g, while the flavonoid content ranged from 11.80 to 24.96 mg Quercetin equivalent/100 g. After the moist-dried heating process, all samples exhibited a significant increase in both phenolic and flavonoid content. The total phenolic content did not significantly differ among the three ratios, whereas the total flavonoid content showed a significant increase with higher honey ratios. The phenolic content ranged from 1785.71 to 1880.19 mg Gallic acid equivalent/100 g, and the flavonoid content ranged from 74.28 to 249.98 mg Quercetin equivalent/100 g. On average, the total phenolic and flavonoid contents increased approximately 8.1 and 8.6 times, respectively, compared to the average values of the samples before the interaction process.

Molaveisi et al. [61] investigated the thermal effect on total phenolic compounds in jujube honey using mild heating treatments ranging from 45 °C to 65 °C over 10 days. The results showed approximately a tenfold increase in total phenolic content. This increase may be attributed to protein structure denaturation, the exposure of active protein sites, the intrinsic degradation of certain antioxidant components, and the formation of Maillard reaction products [61,62]. A study by Daka et al. [63] found that poly-floral honey subjected to thermal treatment at 55 °C and 95 °C for a short duration (30–120 min) exhibited a significant increase in total phenolic and flavonoid content compared to raw honey without heat treatment. This effect can be ascribed to the enhanced bioavailability of specific thermally induced flavonoids or thermostable compounds. Furthermore, the Maillard reaction may contribute to the formation of intrinsically formed and non-dietary antioxidant compounds. Rababah et al. [64] examined the effects of temperature and time on phenolics and flavonoids in crystallized honey, reporting that higher temperatures led to an increase in both phenolic and flavonoid content. However, antioxidant activity decreased, possibly due to the formation of other chemical compounds during heating that are not phenolic and may be more susceptible to degradation. In the context of honey, several studies have shown that phenolic and flavonoid content varies due to factors such as floral or plant origin, climate, geography, bee species, and post-harvest processing. The prominent phenolic compounds found in honey include phenolic acids such as p-coumaric, gallic, ellagic, neochlorogenic, vanillic, protocatechuic, caffeic, and chlorogenic acids, along with flavones (apigenin) and flavonols (quercetin, rutin, myricetin) [5,35,65]. Furthermore, many edible insects, including honeybees (*Apis mellifera*), contain phenolic compounds, with total phenolic content in bee larvae and pupae varying based on their feed source from host plants [66,67].

### 3.4. Changes in Antioxidant Activity Before and After the Moist-Dried Heating Process of HBb-BPs Interacting with Different Honey Concentrations

**Figure 4:**
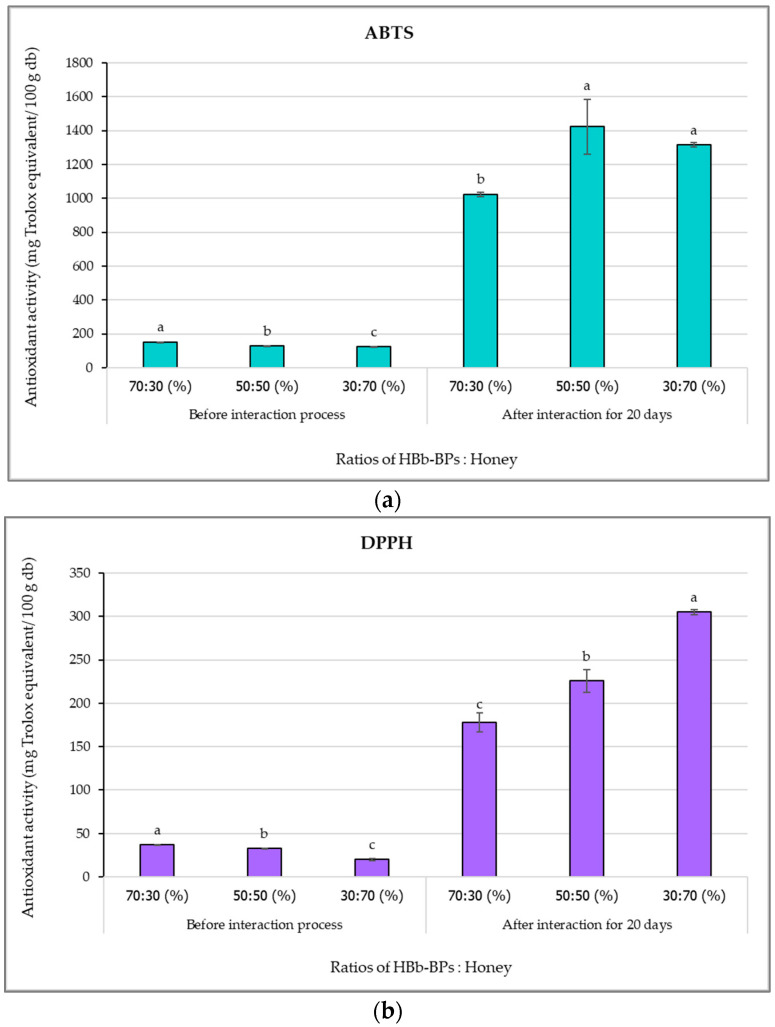

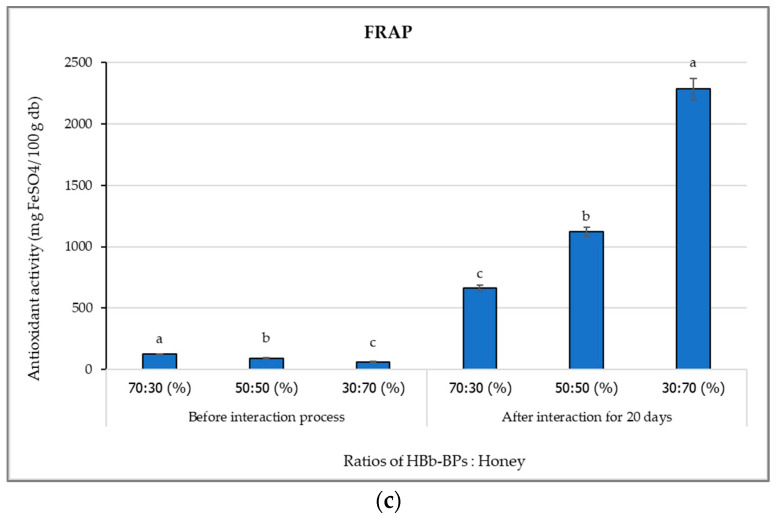
Antioxidant activity by (**a**) ABTS, (**b**) DPPH, and (**c**) FRAP methods of HBb-BPs and honey at ratios of 70:30, 50:50, and 30:70 (% *w*/*w*) before and after interaction through spontaneous aging for 20 days under a moist-dried heating process. Different lowercase letters indicate significant differences among the different honey concentrations before and after the moist-dried heating process (*p* ≤ 0.05).

All samples of HBb-BPs mixed with three different concentrations of honey were tested for antioxidant activity before and after the interaction process using three methods: ABTS, DPPH, and FRAP, as shown in Figure 4. The results indicated that increasing the quantity of added honey significantly reduced the antioxidative capacity of all methods, likely due to a reduction in the percentage of HBb-BPs in the treatment groups before the interaction process. Specifically, the antioxidant activity values of samples without the interaction process ranged from 123.71 to 151.22 mg Trolox equivalent per 100 g for ABTS, from 20.09 to 36.87 mg Trolox equivalent per 100 g for DPPH, and from 62.89 to 126.56 mg FeSO_4_ per 100 g for FRAP.

However, after the interaction process, the antioxidant activity values increased, with higher honey concentrations showing a notable rise in activity levels. For DPPH radical scavenging activity and ferric-reducing antioxidant power, there was a significant increase in antioxidant activity with increasing honey concentration. The ABTS assay also indicated a higher antioxidative capacity after the interaction process. Nevertheless, no significant difference in antioxidant activity was observed between the 50:50 and 30:70 HBb-BPs to honey ratios. The results from the ABTS, DPPH, and FRAP assays showed ranges of 1023.34–1422.47 mg Trolox equivalent per 100 g, 178.11–305.21 mg Trolox equivalent per 100 g, and 661.53–2284.08 mg FeSO_4_ per 100 g, respectively. This indicates an activity increase of 6.8–11.1 times for ABTS, 4.8–15.2 times for DPPH, and 5.2–36.3 times for FRAP.

The antioxidant properties of honey can be influenced by temperature and time, primarily due to the formation of Maillard reaction products such as 5-hydroxymethyl furfuraldehyde (HMF) and sugar–amino acid conjugates, which enhance its antioxidative capacity. Additionally, the phenolic content of honey correlates with increased antioxidant activity during heat treatment, as phenolics act as reducing agents and synergize with other honey components [38,62,63,68]. Peptide and glycoprotein complexes formed via the Maillard reaction also contribute to antioxidant activity, as evidenced by the increase in peptide molecular weight after heating [25,33].

In addition to HBb-BPs, the results indicated that a lower peptide ratio mixed with honey was associated with a decrease in antioxidant activity. This finding aligns with previous research suggesting that honeybee brood contains high levels of antioxidative compounds such as amino acid residues, fatty acids, phenolics, and flavonoids [8]. Furthermore, biopeptides produced from *R. oligosporus* fermentation may also synthesize antioxidant peptides or protein hydrolysates with potent radical scavenging abilities [69]. The presence of D-allulose or psicose after the moist-dried heating process in the HBb-BPs and honey mixture could contribute to scavenging activity against oxygen radicals and reactive oxygen species [41,45]. This rare sugar offers various health-promoting effects, including anti-diabetic, anti-obesity, neuroprotective, anti-hyperglycemic, anti-inflammatory, and antioxidant effects [70]. In the case of mannose, derivatives of deacetylated chitosan-mannose obtained via the Maillard reaction also demonstrated antimicrobial activity against food-borne bacteria, along with antioxidant properties [71].

However, further research is needed to explore the bioactive chemicals from complex natural materials. Advanced techniques, such as liquid chromatography–mass spectrometry (LC-MS) coupled with quadrupole time-of-flight (Q-TOF), offer accurate methods for analyzing and elucidating these complex components [72]. Chemical identification through such methods may help confirm the types of chemical substances that contribute to or influence antioxidant activity.

### 3.5. Correlation Between Each Treatment of HBb-BPs Interacting with Different Honey Concentrations Before and After the Interaction Process on Various Chemical and Antioxidant Properties

**Figure 5:**
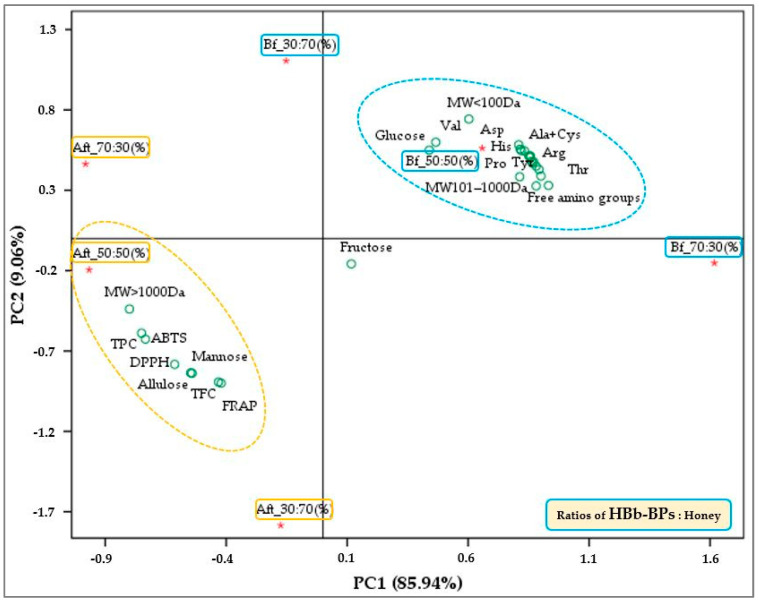
Biplot of principal component analysis (PCA) of HBb-BPs and honey at ratios of 70:30, 50:50, and 30:70 (% *w*/*w*) before (Bf) and after (Aft) interaction through spontaneous aging for 20 days under a moist-dried heating process and their responses of chemical and antioxidant property.

To validate the correlation between the chemical and antioxidant properties of each treatment of HBb-BPs with varying honey concentrations, both before and after the interaction process, principal component analysis (PCA) was performed using the SPSS program (Figure 5). The analysis incorporated data from various chemical determinations, including monosaccharide content, amino acid profiling, available free amino acid groups, peptide chain size distribution, total phenolics, total flavonoids, and antioxidant activities (measured by ABTS, DPPH, and FRAP assays). The PCA identified two components with eigenvalues greater than 1, accounting for 95% of the total variance. Principal component 1 (PC1) explained approximately 85.94% of the variance, while principal component 2 (PC2) accounted for 9.06%. Two main groups emerged from the analysis. The first group consisted of samples with a 50:50 ratio of HBb-BPs to honey before the interaction process. This group was associated with various chemical components, including glucose, free amino acid groups, different types of amino acids, peptides smaller than 100 Da, and peptides between 100 and 1000 Da. The second group comprised samples with a 50:50 ratio of HBb-BPs to honey after the interaction process. This group was associated with mannose, allulose, peptides larger than 1000 Da, total phenolics, total flavonoids, and antioxidant properties as measured by the ABTS, DPPH, and FRAP assays. This analysis confirmed the relationship between certain chemical components and their impact on antioxidant activity. Specifically, antioxidant activities were associated with the presence of allulose, mannose, phenolic compounds, and peptides larger than 1000 Da, which were predominantly observed in samples subjected to the Maillard reaction at a 50:50 ratio of HBb-BPs to honey. Furthermore, the optimal protein-to-sugar ratio for interaction through the Maillard reaction was found to be 1:1, particularly with regard to the effects of higher protein or sugar concentrations on changes in specific chemical substances and bioactivity.

## 4. Conclusions

The moist-dried heating process can induce changes in the chemical characteristics and antioxidant activities of the mixture through the Maillard reaction. Under prolonged thermal and moisture conditions, interactions between HBb-BPs and honey occur, leading to the formation of complex components or structural rearrangements of molecules such as phenolic compounds, Maillard reaction products, and conjugated compounds. These changes may enhance the antioxidant properties of the mixture more effectively than treatments without the moist-dried heating process. Additionally, the ratio of honey, represented by its saccharide portion in the sample, was found to influence the formation of allulose and/or monosaccharide binding with other compounds (amino acids, peptides, phenolics), resulting in increased antioxidant activity. Conversely, higher proportions of honey in the sample led to a decrease in the content of free amino acids, the degree of glycation, and the distribution of larger peptides. Accordingly, this study may suggest that structural modifications in the chemical compounds of honey contribute more significantly to the enhancement of antioxidant activity than biopeptides from bee brood. The optimal radical scavenging ability of the HBb-BPs and honey mixture may be achieved at a 1:1 or 50:50 honey-to-HBb-BPs ratio (% *w*/*w*). However, this study provides preliminary insights into the major chemical components using simple techniques. Future studies employing advanced methods are needed to obtain more accurate results for complex mixed raw materials or ingredients.

## Figures and Tables

**Table 1:** Proximate composition of honeybee brood bio-peptides (HBb-BPs) and honey.

Proximate Composition	HBb-BPs	Honey
Moisture (g/100 g)	42.15 ± 1.25	11.06 ± 0.21
Total protein (g/100 g db)	81.57 ± 0.73	0.54 ± 0.07
Total Carbohydrate (g/100 g db)	2.75 ± 0.52	99.24 ± 0.08
Fat (g/100 g db)	3.60 ± 0.12	0.03 ± 0.01
Fiber (g/100 g db)	5.81 ± 0.10	nd
Ash (g/100 g db)	6.27 ± 0.11	0.19 ± 0.01
Calorific value (kcal/100 g db)	369.65	399.40

Values are means of triplicate determinations; nd indicates not detected; db = dry weight basis.

**Table 2:** Sugar composition before and after interaction through spontaneous aging for 20 days under a moist-dried heating process of HBb-BPs and honey at ratios of 70:30, 50:50, and 30:70 (% *w*/*w*).

Sugars (g/100 g Dry Weight Basis)	Process Conditions	Ratios of HBb-BPs: Honey (%*w*/*w*)		
		70:30	50:50	30:70
Fructose	Before	10.84 ± 0.21 ^cA^	18.06 ± 0.15 ^bA^	25.28 ± 0.11 ^aA^
	After 20 days	4.81 ± 0.24 ^cB^	12.41 ± 0.89 ^bB^	23.84 ± 0.77 ^aA^
Glucose	Before	9.20 ± 0.16 ^cA^	14.83 ± 0.18 ^bA^	22.86 ± 0.42 ^aA^
	After 20 days	0.33 ± 0.11 ^bB^	0.64 ± 0.14 ^bB^	4.42 ± 0.48 ^aB^
Mannose	Before	nd	nd	nd
	After 20 days	0.50 ± 0.11 ^c^	0.83 ± 0.03 ^b^	1.35 ± 0.19 ^a^
Allulose	Before	nd	nd	nd
	After 20 days	0.63 ± 0.05 ^c^	1.19 ± 0.04 ^b^	1.85 ± 0.17 ^a^

Data represented as means ± SD (n = 3); capital letters (A–B) indicate values within each column with different superscript letters were significantly different (*p* ≤ 0.05); lower-case letters (a–c) indicate values within each row with different superscript letters were significantly different (*p* ≤ 0.05); means with the same letters were not significantly different from each other (*p* > 0.05); nd = not detected; Before = before the interaction process; After 20 days = after the interaction process (20 days).

**Table 3:** Amino acid composition before and after interaction through spontaneous aging for 20 days under a moist-dried heating process of HBb-BPs and honey at ratios of 70:30, 50:50, and 30:70 (% *w*/*w*) and the percentage of remaining amino acids.

Amino Acids (mg/100 g Dry Weight Basis)	Process Conditions/ RAA Results	Ratios of HBb-BPs: Honey (%*w*/*w*)		
		70:30	50:50	30:70
Aspartic acid	Before	11.77 ± 0.77 ^aA^	10.55 ± 0.53 ^bA^	8.89 ± 0.18 ^cA^
	After 20 days	3.83 ± 0.72 ^aB^	1.18 ± 0.39 ^bB^	0.79 ± 0.38 ^bB^
	RAA (%)	32.55	11.19	8.89
Threonine	Before	88.73 ± 5.05 ^aA^	50.73 ± 2.06 ^b^	37.49 ± 3.73 ^c^
	After 20 days	5.11 ± 0.05 ^B^	nd	nd
	RAA (%)	5.76	0.00	0.00
Serine	Before	26.15 ± 1.21 ^aA^	22.41 ± 0.03 ^bA^	17.39 ± 0.37 ^cA^
	After 20 days	8.09 ± 0.29 ^aB^	4.36 ± 0.60 ^bB^	3.24 ± 0.05 ^cB^
	RAA (%)	30.94	19.46	18.63
Glutamic acid	Before	163.88 ± 5.26 ^aA^	141.79 ± 0.34 ^bA^	113.90 ± 0.93 ^cA^
	After 20 days	18.32 ± 0.31 ^aB^	11.44 ± 2.69 ^bB^	9.59 ± 0.16 ^bB^
	RAA (%)	11.18	8.07	8.42
Proline	Before	56.01 ± 1.26 ^aA^	52.04 ± 0.17 ^bA^	46.03 ± 0.20 ^cA^
	After 20 days	6.62 ± 0.10 ^aB^	1.49 ± 0.62 ^bB^	0.75 ± 0.08 ^bB^
	RAA (%)	11.82	2.86	1.63
Glycine	Before	20.65 ± 0.66 ^aA^	19.49 ± 0.42 ^bA^	13.42 ± 0.13 ^cA^
	After 20 days	4.12 ± 0.06 ^aB^	0.64 ± 0.01 ^bB^	0.35 ± 0.05 ^bB^
	RAA (%)	19.95	3.28	2.61
Alanine + Cysteine	Before	208.72 ± 4.27 ^aA^	178.26 ± 4.32 ^bA^	137.30 ± 2.06 ^cA^
	After 20 days	63.83 ± 2.04 ^aB^	9.66 ± 1.14 ^bB^	6.80 ± 0.66 ^bB^
	RAA (%)	30.58	5.42	4.95
Valine	Before	212.75 ± 8.25 ^aA^	183.22 ± 1.77 ^bA^	143.27 ± 2.01 ^cA^
	After 20 days	196.87 ± 0.09 ^aB^	125.29 ± 2.34 ^bB^	83.82 ± 2.05 ^cB^
	RAA (%)	92.53	68.38	58.50
Methionine	Before	8.82 ± 1.20 ^a^	7.65 ± 0.11 ^b^	5.83 ± 0.59 ^c^
	After 20 days	nd	nd	nd
	RAA (%)	0.00	0.00	0.00
Isoleucine	Before	23.44 ± 0.47 ^aA^	21.01 ± 0.67 ^b^	16.16 ± 0.89 ^c^
	After 20 days	3.18 ± 0.54 ^B^	nd	nd
	RAA (%)	13.56	0.00	0.00
Leucine	Before	51.37 ± 1.31 ^aA^	45.91 ± 4.47 ^b^	36.66 ± 4.77 ^c^
	After 20 days	1.45 ± 0.09 ^B^	nd	nd
	RAA (%)	2.82	0.00	0.00
Tyrosine	Before	87.90 ± 9.13 ^a^	51.03 ± 6.99 ^b^	51.75 ± 1.93 ^b^
	After 20 days	nd	nd	nd
	RAA (%)	0.00	0.00	0.00
Phenylalanine	Before	168.82 ± 4.99 ^a^	146.14 ± 1.99 ^b^	121.37 ± 0.09 ^c^
	After 20 days	nd	nd	nd
	RAA (%)	0.00	0.00	0.00
Histidine	Before	42.79 ± 3.20 ^aA^	36.02 ± 0.32 ^bA^	35.64 ± 4.32 ^bA^
	After 20 days	8.79 ± 1.17 ^aB^	4.27 ± 0.55 ^bB^	3.22 ± 0.06 ^bB^
	RAA (%)	20.54	11.86	9.04
Lysine	Before	35.83 ± 0.41 ^aA^	31.60 ± 2.41 ^bA^	25.00 ± 1.83 ^cA^
	After 20 days	5.08 ± 0.16 ^aB^	1.04 ± 0.45 ^bB^	0.89 ± 0.06 ^bB^
	RAA (%)	14.18	3.29	3.56
Arginine	Before	7.46 ± 2.09 ^a^	5.53 ± 0.25 ^b^	4.73 ± 0.58 ^b^
	After 20 days	nd	nd	nd
	RAA (%)	0.00	0.00	0.00
TAA	Before	1215.40	1003.89	815.53
	After 20 days	423.72	221.63	151.36
	RAA (%)	26.76	15.88	13.42

Data represented as means ± SD (n = 3); capital letters (A–B) indicate values within each column with different superscript letters were significantly different (*p* ≤ 0.05); lower-case letters (a–c) indicate values within each row with different superscript letters were significantly different (*p* ≤ 0.05); means with the same letters were not significantly different from each other (*p* > 0.05); nd = not detected; TAA = Total amino acids indicated sum of 17 amino acids; RAA = Percentage of remaining amino acid content after the interaction process compared to before the interaction process; Before = before the interaction process; After 20 days = after the interaction process (20 days).

## Data Availability

The original contributions presented in the study are included in the article. Further inquiries can be directed to the corresponding authors.
